# False arrhythmia alarms can be reduced by algorithm improvements while the magnitude of the reduction is affected by alarm settings

**DOI:** 10.1186/cc14235

**Published:** 2015-03-16

**Authors:** M Kaski, J Vanhatalo, S Treacy, H Viertio-Oja

**Affiliations:** 1 GE Healthcare, Helsinki, Finland; 2 GE, Milwaukee, WI, USA

## Introduction

The ECRI Institute has identified alarm fatigue as the number one health technology hazard [1]. A recent study on 461 ICU patients investigated 2,558,760 alarms [2]. In total, 88.8% of the annotated 12,671 arrhythmia alarms were false positives (FPs). It was concluded that the excessive number of alarms is 'a complex interplay of inappropriate user settings, patient conditions, and algorithm deficiencies'. Nine conditions causing alarms, four of which were ECG algorithm related, were reported [2]. In this study, we investigated a new algorithm in which improvements targeting three of the reported four ECG-related conditions were implemented: low amplitude QRS; wide QRS; nonactionable ventricular tachycardia (VT).

## Methods

The false alarm rate of the new algorithm (GE Carescape, 2012) was compared with that of the algorithm evaluated in the study (GE Solar, 2003) [2] on the collected ECG waveform data. User settings such as QRS detection sensitivity (high/normal) were not available. Therefore, normal sensitivity was assumed for both versions. With the old algorithm, 10 patients with low QRS amplitudes gave a significantly higher number of FPs than were reported [2]. For those patients, both sensitivity modes were tested with the old algorithm. Sixty-six percent of patients with a pacemaker did not have the pacemaker mode selected [2]. Outlier patients in which false alarms were due to user settings (20 patients with a pacemaker) or patient condition (four patients with a bundle branch block) rather than algorithm deficiency were excluded.

## Results

Improved algorithm resulted in 66% reduction of FP alarms. When using the high-sensitivity mode for the 10 patients with low QRS, FP reduction was 18%. No compromises regarding detection of true events were found. The 24 outlier patients contributed to 81.3% of FP alarms. The algorithm changes responsible for the reduced FPs were: adaptive threshold for low amplitude QRS detection; QRS filter with an extended frequency range; management of VT alarm priorities.

## Conclusion

A majority of the FPs was linked to user settings and patient conditions. The algorithm changes resulted in a clear reduction of ECG algorithm-related FP alarms, while the magnitude of the reduction depends strongly on the settings at the bedside.
